# TAOK1-mediated regulation of the YAP/TEAD pathway as a potential therapeutic target in heart failure

**DOI:** 10.1371/journal.pone.0308619

**Published:** 2024-08-09

**Authors:** Jiani Zhou, Chaoqun Wu, Miaohui Zhao

**Affiliations:** Department of General Practice, Ningbo Medical Treatment Center Li Huili Hospital, Affiliated to Ningbo University, Ningbo, Zhejiang, China; Jordan University of Science and Technology Faculty of Medicine, JORDAN

## Abstract

**Background:**

This study aimed to determine the roles of interleukin (IL)-17, TAO kinase 1 (TAOK1), and NOD-like receptor protein 3 (NLRP3) in cardiomyocyte pyroptosis and proliferation.

**Methods:**

The IL-17-treated H9C2 cells were used as *in vitro* heart failure (HF) models. These cells were subjected to *TAOK1* overexpression or knockdown and treated with BMS-986299 (NLRP3 inflammasome agonist), MCC950 (NLRP3 inflammasome inhibitor), or verteporfin (Yes-associated protein [YAP] inhibitor). Thereafter, their pyroptosis, proliferative capacity, and gene and protein expression levels were detected. Doxorubicin-induced HF rats were used as *in vivo* models and subjected to *TAOK1* overexpression. Thereafter, their myocardial pathology, NLRP3 inflammasome-mediated pyroptosis, and YAP/TEAD pathway function were evaluated.

**Results:**

IL-17 treatment increased the pyroptosis and decreased the proliferative capacity of H9C2 cells. Additionally, IL-17 treatment inducedto the activation of the NLRP3 inflammasomes and inhibition of the YAP/TEAD pathway in the H9C2 cells. Moreover, the IL-17-mediated effects on the H9C2 cells were alleviated by *TAOK1* overexpression and augmented by *TAOK1* knockdown. Furthermore, treatment with BMS-986299 or verteporfin affected the pyroptosis, proliferative capacity, and NLRP3 inflammasome activation of the H9C2 cells independently of TAOK1 expression. In the doxorubicin-induced HF rat model, *TAOK1* overexpression mitigated myocardial injury, suppressed NLRP3 inflammasome pathway activation, and restored the YAP/TEAD pathway activity.

**Conclusion:**

TAOK1 played a crucial role in regulating IL-17-mediated increase in the pyroptosis and decrease in the proliferation of cardiomyocytes by regulating the activities of the NLRP3 inflammasomes and the YAP/TEAD pathway.

## 1 Introduction

Heart failure (HF) is a complex condition characterized by impaired ventricular filling or ejection capacity due to various cardiac structural or functional abnormalities. HF leads to a rational remodeling of cardiomyopathy and a decline in the heart’s pumping function [1, 2]. There has been a steady increase in the prevalence of HF due to the increase in the incidence of cardiovascular conditions, such as hypertension and coronary heart disease [3]. HF remains a chronic progressive disease despite several advancements in its medical treatment that have improved the survival rate and extended the survival time of HF patients. HF progression may injure the myocardium, thereby impairing the heart’s pumping function. This can trigger an automatic stimulation of the sympathetic nervous system and the renin–angiotensin–aldosterone system, resulting in subsequent myocardial remodeling, fibrosis, and inadequate arterial blood supply or venous congestion, ultimately resulting in tissue and organ dysfunction [4]. Therefore, a comprehensive analysis of HF pathogenesis can provide novel therapeutic strategies to improve the prognosis of HF patients.

Interleukin (IL)-17A is primarily produced by inflammatory cells, such as helper T cells, activated macrophages, and neutrophils. It has been associated with several chronic inflammatory and autoimmune diseases, including Psoriasis [5, 6]. Additionally, it contributes to the advancement of several heart diseases, including dilated cardiomyopathy [7], myocardial infarction [8], and atherosclerosis [9]. Moreover, IL-17A has been demonstrated to be associated with NF-κB-mediated cardiac remodeling in HF [10]. However, the underlying mechanism through which IL-17A affects HF progression, especially by regulating cardiomyocyte autophagy and inflammation, remains unclear.

TAO kinase 1 (TAOK1), a member of the sterile 20 kinase subfamily, plays a critical role in inducing morphological changes during apoptosis via the activation of the c-Jun NH2-terminal kinase (JNK)/mitogen-activated protein kinases (MAPK) pathway [11]. Additionally, TAOK1 regulates the initiation of DNA damage through the JNK/P38 MAPK pathway [12]. In addition, TAOK1 is associated with neurodevelopmental disorders [13, 14] and cancers, including Non-Small-Cell Lung Cancer Cells, hepatocellular carcinoma et al [15, 16]. Furthermore, TAOK1 has been reported to reduce the IL-17A-induced expression of inflammatory cytokines and chemokines [17]. However, its role in HF progression via the regulation of IL-17A remains unexplored.

Pyroptosis, a subclass of programmed cell death, is primarily characterized by the activation of caspase 1 and the release of pro-inflammatory factors [18]. Previously, bacterial and viral infections were implicated in the activation of pyroptosis; however, increasing evidence has revealed that non-infectious diseases, such as neurodegenerative disorders and atherosclerosis, are also associated with pyroptosis [19]. The NLRP3 inflammasome-mediated pyroptosis has received widespread attention, and it has been found to play a crucial role in the pathogenesis of cardiovascular diseases [20]. A study demonstrated that NLRP3/gasdermin D (GSDMD)-mediated pyroptosis is crucial for HF development in mice models of HF with preserved ejection fraction (HFpEF) [21]. However, the mechanisms underlying NLRP3 inflammasome-mediated pyroptosis in HF remain to be elucidated. Liu et al. [22] found that IL-17A promotes the activation of the NLRP3 inflammasomes in lung cancer cells, consequently enhancing their malignant behavior [22]. Similarly, Zhang et al. [23] demonstrated that IL-17A elevates IL-1β levels by activating NLRP3 inflammasomes in primary human retinal pigment epithelial cells. Based on the previous reports, we hypothesized that TAOK1 may hinder the activation of NLRP3 inflammasomes to exert its inhibitory effects on inflammatory responses. The Yes-associated protein (YAP)/transcriptional enhanced associate domain (TEAD) pathway is considered to play a crucial role in cardiovascular diseases, wherein YAP, in association with transforming growth factor (TGF)-β, plays a fibrotic therapeutic role [24]. Moreover, von Gise et al. [25]reported that YAP stimulates the proliferation of cardiomyocytes. Another study found that YAP inhibitors alleviate cardiac fibrosis and functional impairment in cardiovascular diseases [26].

**Fig 1:**
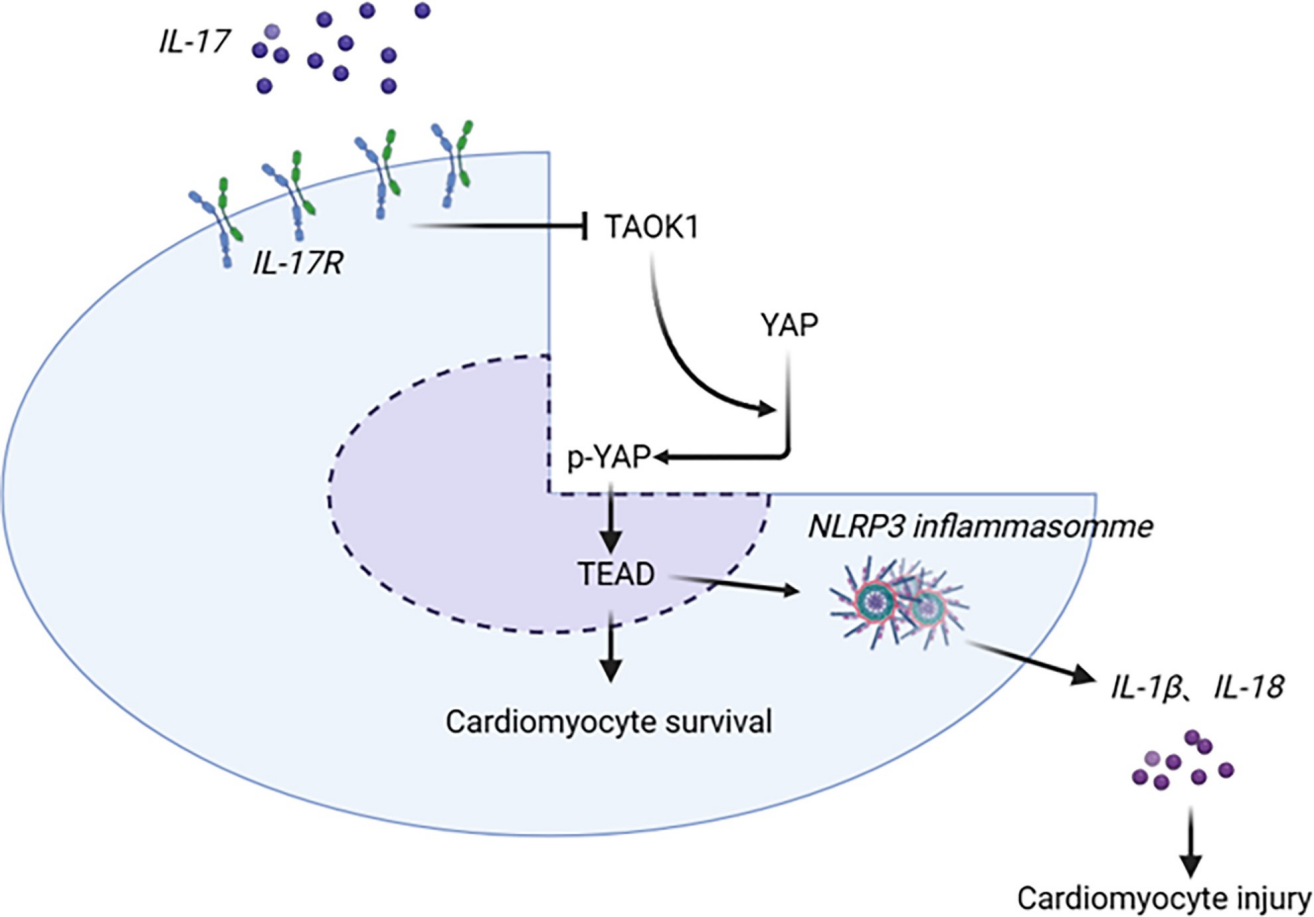
Schematic representation of the role of TAOK1 in IL-17-mediated pyroptosis and proliferation of cardiomyocytes. TAOK1 affects IL-17-mediated pyroptosis and proliferation of cardiomyocytes by regulating the activity of the NLRP3 inflammasome and the YAP/TEAD pathway.

Our study aimed to investigate the roles of IL-17A and TAOK1 on cardiomyocyte pyroptosis, proliferation, and NLRP3 inflammasome and YAP/TEAD pathway activation. However, further experimental analyses are required to validate the effects of NLRP3 inflammasomes and the YAP/TEAD pathway on cardiomyocyte proliferation and pyroptosis (Fig 1). Our results enhance our understanding of HF pathogenesis and provide the scientific basis for more comprehensive research in the future.

## 2 Materials and methods

### 2.1 Cell culture

The H9C2 cells (Saiye Biotechnology, Guangzhou, China) were cultivated in Dulbecco’s modified Eagle medium (Gibco, Thermo Fisher Scientific, MA, USA) supplemented with 10% fetal bovine serum (Sigma) at 37°C and 5% CO_2_. The culture medium was renewed every alternate day, and the cells were passaged upon reaching 70–80% confluency.

### 2.2 Cell treatment

The *TAOK1*-siRNA and negative control (NC)-siRNA were obtained from GenePharma (Suzhou, China), and the empty vector, *TAOK1*-overexpressing plasmid (OE-*TAOK1*), and *NLRP3*-overexpressing plasmid (OE-*NLRP3*) were obtained from Invitrogen (Carlsbad, CA, USA). The H9C2 cells (1X10^6^ cells) were seeded in a six-well plate and exposed to 10, 20, or 40 ng/mL of IL-17A [27]. Thereafter, the cells were transfected with *TAOK1*-siRNA or OE-*TAOK1* plasmid using Lipofectamine 3000 (Invitrogen), according to the manufacturer’s instructions. Additionally, some H9C2 cells were treated with BMS-986299 (NLRP3 antagonist, Macklin, Shanghai, China), MCC950 (NLRP3 inhibitor, Macklin, Shanghai, China), or verteporfin (YAP inhibitor, MedChemExpress, USA).

### 2.3 Flow cytometry (FC) analysis

The H9C2 cells were incubated for 48 h with FLICA working solution from the FAM FLICA™ Caspase-1 kit (ICT097, Bio-Rad). Thereafter, the cells were washed with cell wash buffer and incubated with accutase for 10 min at 37°C. Subsequently, the cells were transferred to centrifuge tubes and treated with PI staining solution. FC assay was conducted to assess H9C2 cell pyroptosis by BD Biosciences (San Diego, CA, USA).

### 2.4 EdU staining

H9C2 cells were seeded in 6-well plates, added with 10 μM EdU working solution, and incubated at 37°C for 90 minutes. Subsequently, the cells were thoroughly washed, and 0.3% Triton-X solution was added dropwise and incubated at room temperature for 10 minutes. According to the instructions, EdU Click working solution was prepared and cells were incubated with EdU Click working solution at room temperature for 30 minutes in the dark. After washing, DAPI staining solution was added and the cells were observed under a fluorescence microscope for cell proliferation. The EdU staining kit was purchased from Beyotime Biotechnology Co., Ltd., (C0075S, Shanghai, China).

### 2.5 Cell counting kit (CCK)-8 analysis

The H9C2 cells were seeded in 96-well plates (1×10^4^ cells/well) and incubated overnight at 37°C and 5% CO_2_. After adherence, the cells were treated with 1, 5, or 10 ng/mL IL-17A for 48 h. Subsequently, the cells were incubated with 10 μL of CCK-8 reagent (Dojindo, Kumamoto, Japan) for 3 h. The optical density value of each well was measured at 450 nm using an automatic microplate reader (Thermo Fisher Scientific, multi-scan MK3).

### 2.6 Quantitative reverse transcription-polymerase chain reaction (qRT-PCR) analysis

Total RNA was extracted from the H9C2 cells using TRIzol reagent (Invitrogen, MA, USA) and then converted into complementary DNA (cDNA) using a reverse transcription kit (Takara, Tokyo, Japan). The resulting cDNA was used as a template for PCR amplification using the SYBR Green qPCR Master Mix (DBI Bioscience). The relative gene expression was determined using the 2^*-ΔΔCt*^ method. GAPDH was used as internal reference gene.

### 2.7 Western blotting (WB) analysis

The H9C2 cells were lysed on ice using RIPA buffer with protease inhibitors (Beyotime, China) for protein extraction, and the proteins were quantified using the bicinchoninic acid method. Subsequently, 50 μg of protein from each sample was separated using 10% sodium dodecyl sulfate–polyacrylamide gel electrophoresis and transferred onto polyvinylidene fluoride membranes (Millipore) at 200 mA. Thereafter, the membranes were blocked with 5% skim milk powder solution for 2 h and then incubated overnight with primary antibodies (1:2000; Abcam) at 4°C. Subsequently, the membranes were incubated with secondary antibodies (1:2000; Abcam) for 1 h at 4°C. Lastly, the membranes were washed and treated with electrochemiluminescence reagent (Millipore), and the images were analyzed using the Image J software.

### 2.8 Immunofluorescence (IF) assay

The H9C2 cells were placed on coverslips and allowed to adhere. Subsequently, the cells were fixed with 4% paraformaldehyde and then incubated overnight with anti-NLRP3 antibodies at 4°C. Thereafter, the cells were incubated with fluorescently labeled secondary antibodies at room temperature for 60 min. Lastly, the cells were stained with 4’,6-diamidino-2-phenylindole and visualized using a laser confocal microscope.

### 2.9 Enzyme-linked immunosorbent assay (ELISA)

The H9C2 cell culture samples were centrifuged at 3000 g for 10 min, and the supernatants were collected. Thereafter, the IL-1β and IL-18 levels in the samples were quantified using the corresponding ELISA kits (Elabscience, Hubei, China), according to the manufacturer’s instructions.

### 2.10 Rat HF model

A total of 32 Sprague–Dawley rats (200–250 g) were provided by Ningbo University Animal Laboratory Center and randomly divided into four groups (n = 8/group), namely the sham group, HF group, vector group, and TAOK1 group. The rats in the HF, vector, and TAOK1 groups were intraperitoneally injected with 20 mg/kg/week doxorubicin for 6 weeks to establish the HF model [28], while the rats in the sham group were injected with an equal volume of saline. The rats in the vector and TAOK1 groups were injected with 150 μL of AAV9-empty vector or AAV9-*TAOK1* vector (2 × 10^11^ GC/mL; Genechem Co., Ltd., Shanghai, China), respectively, via tail vein injection, 4 weeks prior to HF modeling. The rats were euthanized by intraperitoneal injection of 150 mg/kg sodium pentobarbital, and the serum and cardiac tissues were obtained for subsequent analyses. Animal experiments were approved by the Experimental Animal Ethics Committee of Ningbo University (Application number: 12813; Zhejiang, China).

### 2.11 Hematoxylin and eosin (H&E) staining

The cardiac tissues were fixed, paraffin-embedded, and sectioned. Thereafter, the sections were dehydrated, deparaffinized, and subjected to H&E staining (Guangzhou ServiceBio, Guangzhou, China). Lastly, the slides were washed and covered with coverslips using neutral resin for microscopic examination.

### 2.12 Terminal deoxynucleotidyl transferase dUTP nick end labeling (TUNEL) assay

The cardiac tissue sections were dehydrated, deparaffinized, and treated with proteinase K. Thereafter, the sections were washed and treated with hydrogen peroxide solution to block endogenous peroxidase activity. Subsequently, the sections were incubated in the dark with a biotin-labeled solution for 1 h at 37°C, and the reaction was terminated by adding the stopping solution. Thereafter, the sections were treated with streptavidin–horseradish peroxidase working solution followed by DAB chromogen solution. Afterwards, the sections were washed and covered with coverslips using neutral resin for microscopic examination. The TUNEL detection kit was obtained from Beyotime Bio (Shanghai, China).

### 2.13 Immunohistochemistry staining

The cardiac tissue sections were dehydrated, deparaffinized, and then subjected to antigen retrieval and endogenous peroxidase blocking. Thereafter, the sections were blocked using the serum samples for 30 min at room temperature. Subsequently, the sections were incubated with anti-NLRP3 antibodies (ServiceBio) and corresponding secondary antibodies (K5007, DAKO) and then developed with DAB staining solution. The samples were counterstained with hematoxylin, dehydrated, and mounted with coverslips for microscopic examination.

### 2.14 Statistical analysis

All the experiments were conducted in triplicates, and the data are presented as mean ± standard deviation. Statistical analysis was conducted using one-way analysis of variance on the SPSS 22.0 software. A *p*-value of < 0.05 was considered statistically significant.

## 3 Results

### 3.1 Effects of IL-17 on the pyroptosis, proliferative capacity, and TAOK1 expression of the H9C2 cells

**Fig 2:**
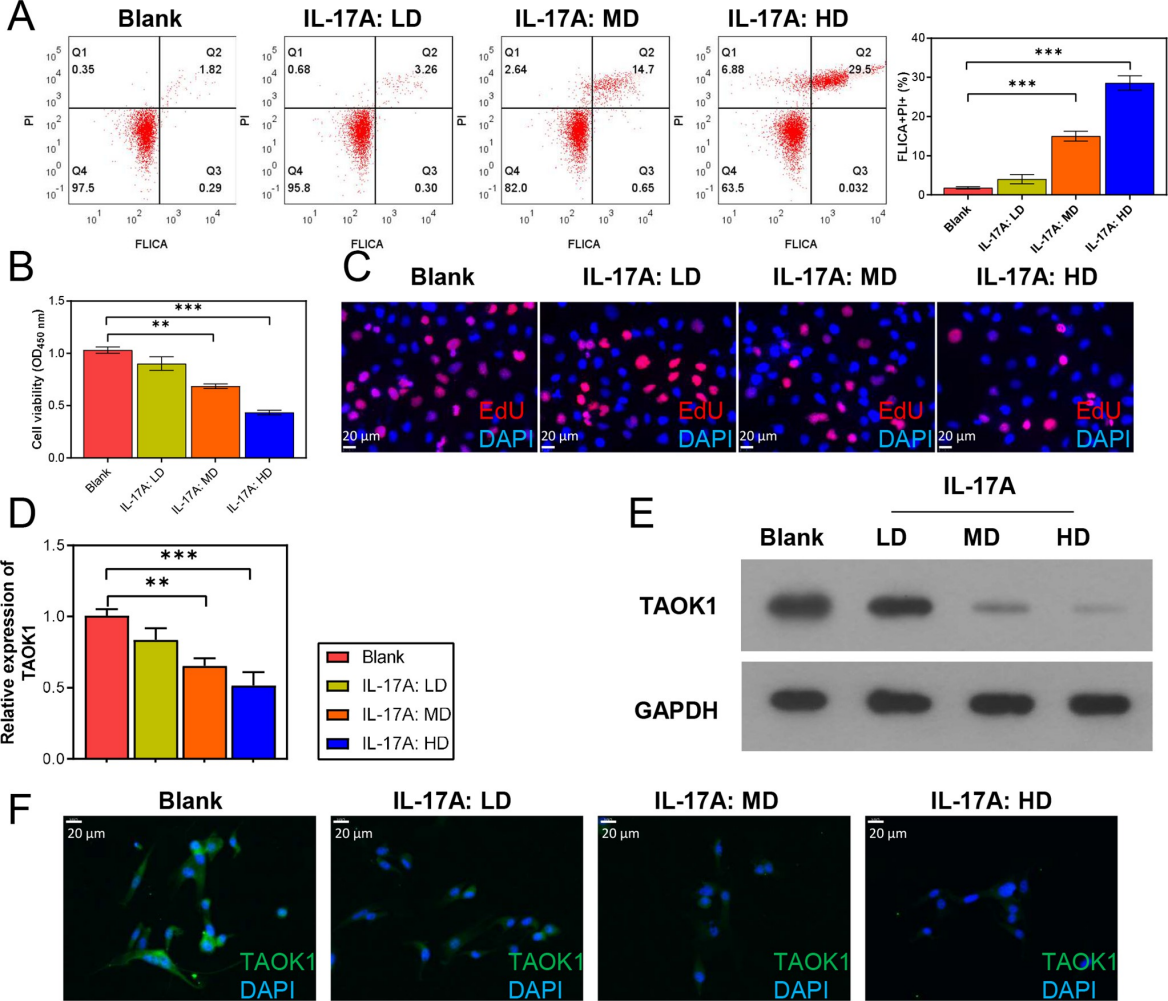
Effects of IL-17 on the pyroptosis, proliferative capacity, and TAOK1 expression of H9C2 cells. The H9C2 cells were treated with 10, 20, or 40 ng/mL of IL-17, and their pyroptosis, proliferation capacity, and TAOK1 expression were determined. (A) FC analysis was used to detect the pyroptosis of H9C2 cells. (B, C) CCK-8 (B) and EdU staining (C) assays were employed to detect the proliferative capacity of H9C2 cells. (D–F) qRT-PCR (D), WB (E), and IF (F) analyses were used to detect the expression level and localization of TAOK1 in the H9C2 cells. **P < 0.01, ***P < 0.001.

To explore the effects of IL-17 on cardiomyocytes, the H9C2 cells were exposed to varying concentrations of IL-17, and their pyroptosis, proliferative capacity, and TAOK1 expression were determined. FC analysis revealed that an increase in IL-17 concentration was associated with a gradual increase in H9C2 cell pyroptosis (FLICA+/PI+; Fig 2A). Meanwhile, CCK-8 and EdU staining assays showed that the proliferative ability of the H9C2 cells gradually decreased with an increase in IL-17 levels (Fig 2B and 2C). Additionally, qRT-PCR, WB, and IF analyses revealed that TAOK1 expression in the H9C2 cells decreased gradually with an increase in IL-17 concentration (Fig 2D–2F).

### 3.2 TAOK1 suppressed IL-17-mediated NLRP3 inflammasome activation by modulating the YAP/TEAD pathway in the H9C2 cells

**Fig 3:**
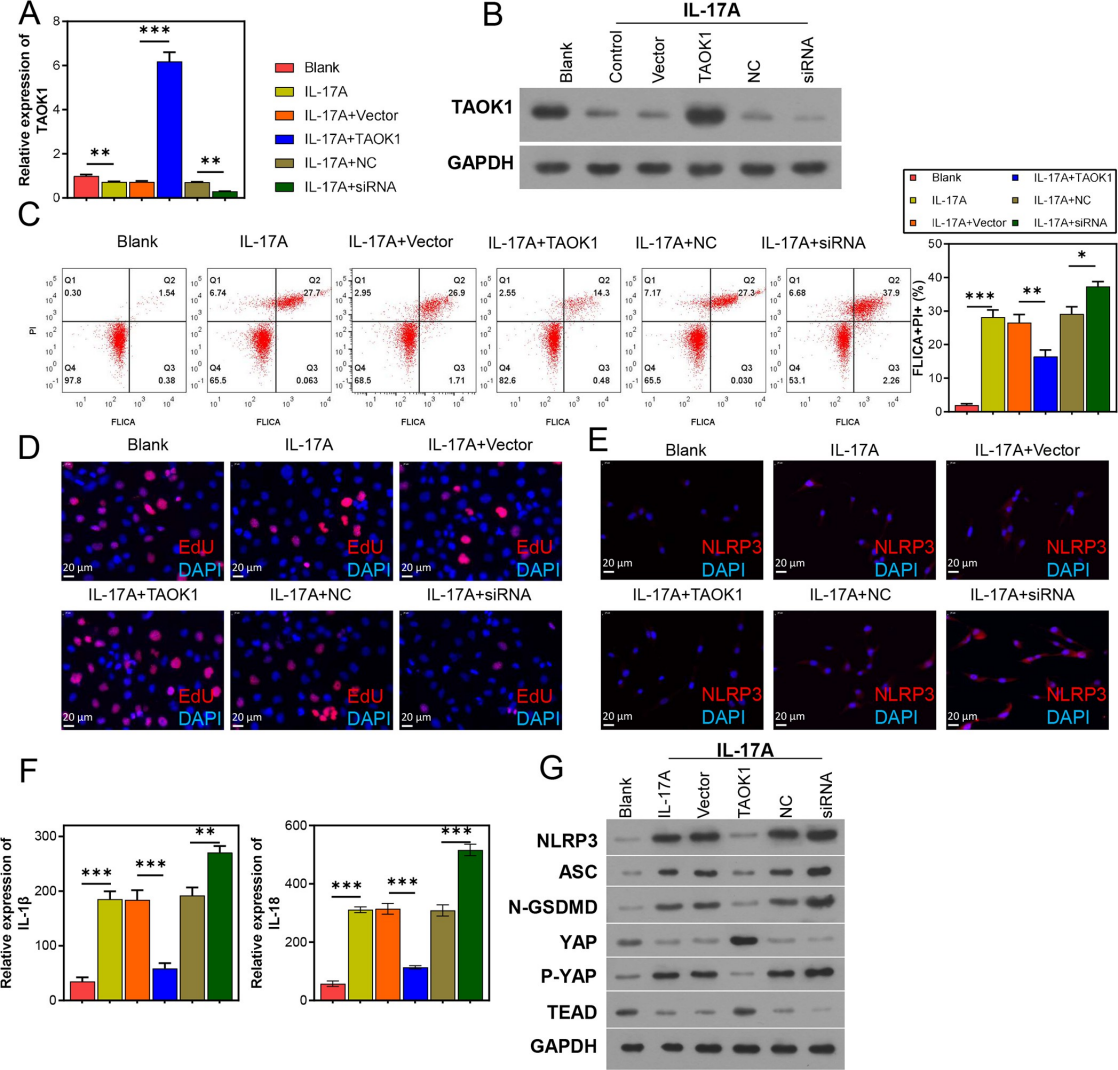
Effects of TAOK1 expression in IL-17 treated H9C2 cells. The IL-17-treated H9C2 cells were subjected to *TAOK1* overexpression and knockdown, and their pyroptosis, proliferative capacity, and gene and protein expression levels were determined. (A, B) qRT-PCR (A) and WB (B) analyses were used to detect TAOK1 expression in the H9C2 cells. (C) FC analysis was used to detect the pyroptosis of H9C2 cells. (D) EdU staining assay was used to detect the proliferative capacity of H9C2 cells. (E) IF analysis was used to detect NLRP3 expression in the H9C2 cells. (F) ELISA was used to detect IL-1β and IL-18 levels in the supernatant of H9C2 cells. (G) WB assay was used to detect the expression of proteins associated with NLRP3 inflammasomes and YAP/TEAD pathway activation in the H9C2 cells. **P < 0.01, ***P < 0.001.

IL-17 treatment led to the suppression of TAOK1 in H9C2 cells (Fig 3A and 3B). The results revealed that *TAOK1* overexpression significantly attenuated and *TAOK1* knockdown promoted the IL-17A-induced increase in the proportion of FLICA+/PI+ H9C2 cells (Fig 3C). In addition, *TAOK1* overexpression attenuated, while *TAOK1* knockdown further exacerbated the IL-17A-induced decrease in H9C2 cell proliferation (Fig 3D). Furthermore, IL-17 elevated NLRP3, ASC, and N-GSDMD levels and significantly upregulated IL-18 and IL-1β levels (Fig 3E–3G), and this upregulation was augmented by *TAOK1* knockdown and suppressed by *TAOK1* overexpression (Fig 3D–3F). Further analysis revealed that IL-17 inhibited the YAP/TEAD pathway, which was exacerbated by *TAOK1* knockdown and attenuated by *TAOK1* overexpression (Fig 3G).

### 3.3 *TAOK1* knockdown promoted NLRP3 inflammasome activation in the H9C2 cells

**Fig 4:**
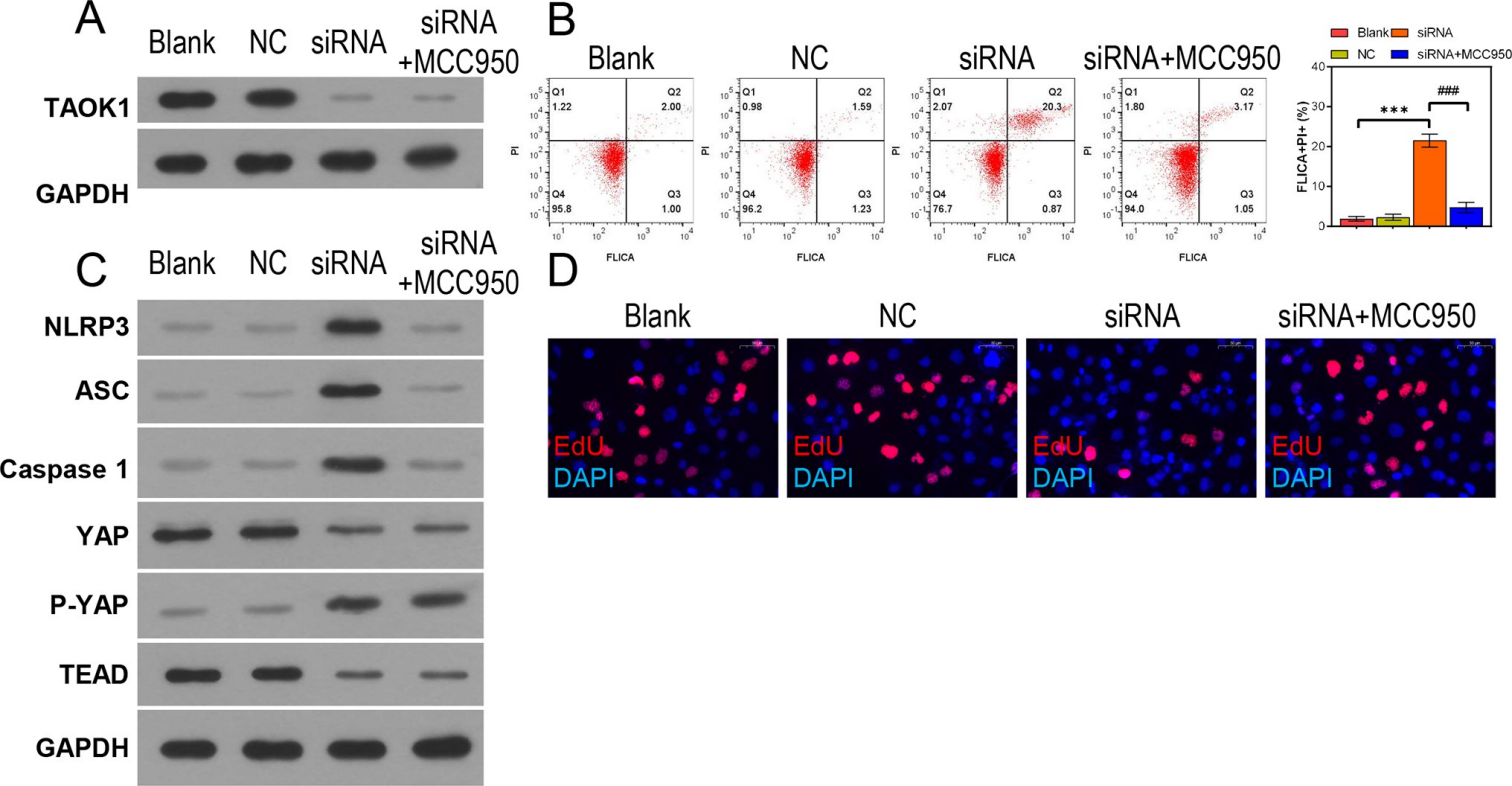
*TAOK1* knockdown promoted NLRP3 inflammasome activation in the H9C2 cells. The H9C2 cells were subjected to *TAOK1* knockdown and MCC950 treatment, and their pyroptosis, proliferative capacity, and gene and protein expression levels were determined. (A) WB assay was used to detect TAOK1 expression in the H9C2 cells. (B) FC analysis was used to detect the pyroptosis of H9C2 cells. (C) WB assay was used to detect the expression of NLRP3 inflammasome- and YAP/TEAD pathway-related proteins in the H9C2 cells. (D) IF analysis was used to detect NLRP3 expression in the H9C2 cells. (E) EdU staining assay was used to detect the proliferative capacity of H9C2 cells. *P < 0.05, **P < 0.01, ***P < 0.001.

To further elucidate the role of TAOK1 in cardiomyocytes, we detected the effects of *TAOK1* knockdown and MCC950 incubation on *TAOK1* expression in the H9C2 cells. The results indicated that *TAOK1* knockdown significantly reduced *TAOK1* expression in H9C2 cells, which remained unaffected by MCC950 treatment (Fig 4A). FC analysis further revealed that *TAOK1* knockdown significantly increased and MCC950 treatment significantly decreased the proportion of FLICA+/PI+ H9C2 cells (Fig 4B). Additionally, *TAOK1* knockdown induced an increase in the expression of NLRP3 inflammasome-associated proteins, CASPASE 1 and ASC; however, MCC950 treatment decreased the expression of these proteins in H9C2 cells (Fig 4C). Notably, *TAOK1* knockdown inhibited the YAP/TEAD pathway, as indicated by the upregulation of p-YAP expression and downregulation of TEAD expression. In contrast, MCC950 treatment did not significantly affect the YAP/TEAD pathway (Fig 4C). Lastly, EdU staining experiments revealed that *TAOK1* knockdown inhibited and MCC950 treatment promoted the proliferation of H9C2 cells (Fig 4D).

### 3.4 TAOK1 regulated the proliferation and NLRP3 inflammasome-mediated pyroptosis of the H9C2 cells

**Fig 5:**
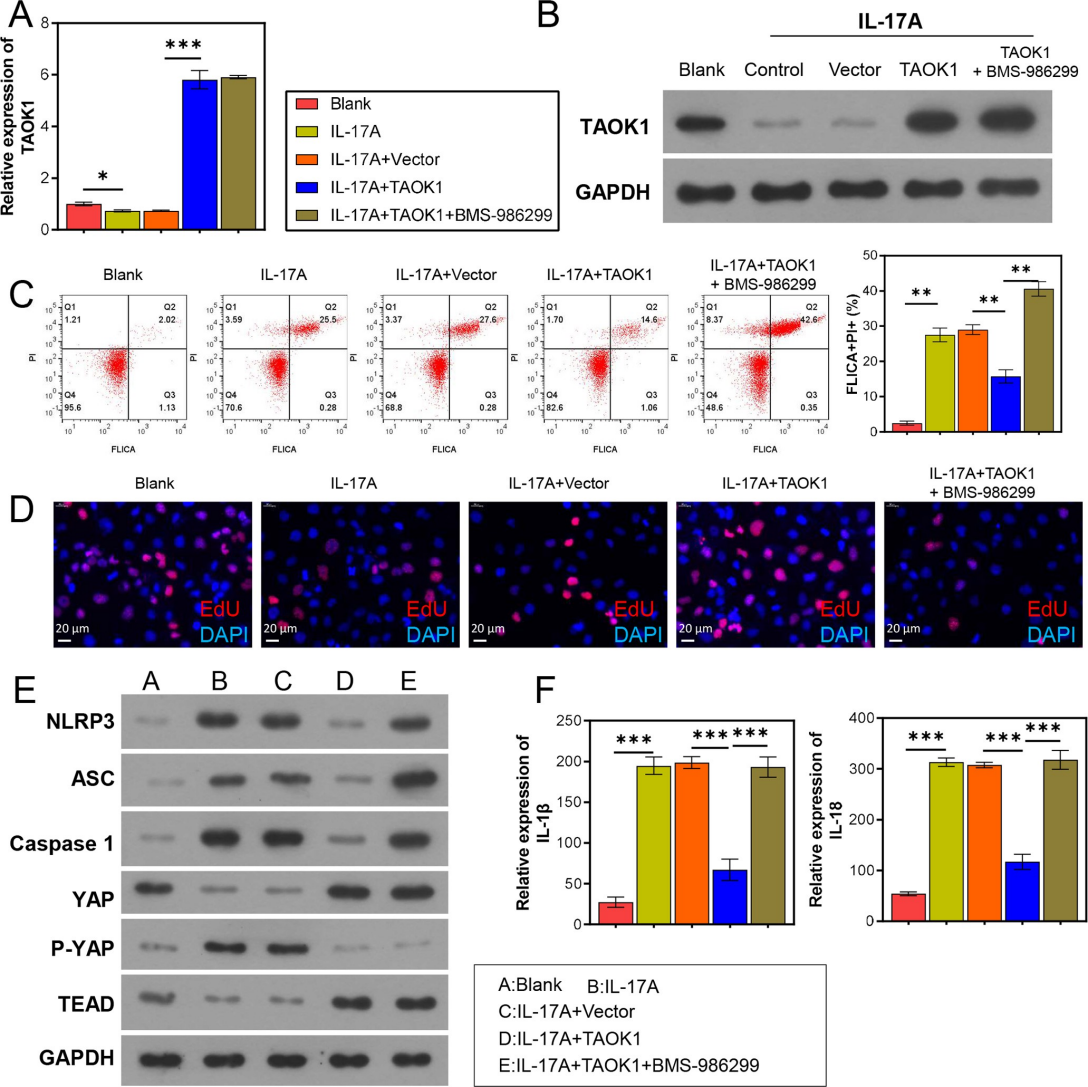
Role of NLRP3 inflammasome complex in the IL-17-induced adverse effects on the H9C2 cells. The H9C2 cells were subjected to *TAOK1* overexpression and BMS-986299 treatment, and their pyroptosis, proliferative capacity, and gene and protein expression levels were determined. (A, B) qRT-PCR (A) and WB (B) analyses were used to detect TAOK1 expression in the H9C2 cells. (C) FC analysis was used to detect the pyroptosis of H9C2 cells. (D) EdU staining assay was used to detect the proliferative capacity of H9C2 cells. (E) WB assay was used to detect the expression of NLRP3 inflammasome-related proteins in the H9C2 cells. (F) ELISA was used to detect IL-1β and IL-18 levels in the supernatant of H9C2 cells. *P < 0.05, **P < 0.01, ***P < 0.001.

To elucidate the role of NLRP3 inflammasomes in the IL-17-treated H9C2 cells, the cells were administered BMS-986299, and their pyroptosis, proliferative capacity, and TAOK1 expression were determined. The results revealed that BMS-986299 treatment did not alter the expression of *TAOK1* in the IL-17-treated H9C2 cells (Fig 5A and 5B); however, it reversed the effects of *TAOK1* overexpression on the pyroptosis and proliferative capacity of the IL-17-treated H9C2 cells (Fig 5C and 5D). Additionally, the BMS-986299 treatment attenuated the inhibitory effects of *TAOK1* overexpression on NLRP3 inflammasomes, resulting in the upregulation of IL-1β and IL-18 (Fig 5E and 5F). However, it did not affect the YAP/TEAD pathway in the IL-17-treated H9C2 cells (Fig 5E and 5F).

### 3.5 TAOK1 inhibited the NLRP3 inflammasome-mediated pyroptosis of the H9C2 cells by regulating the YAP/TEAD pathway

**Fig 6:**
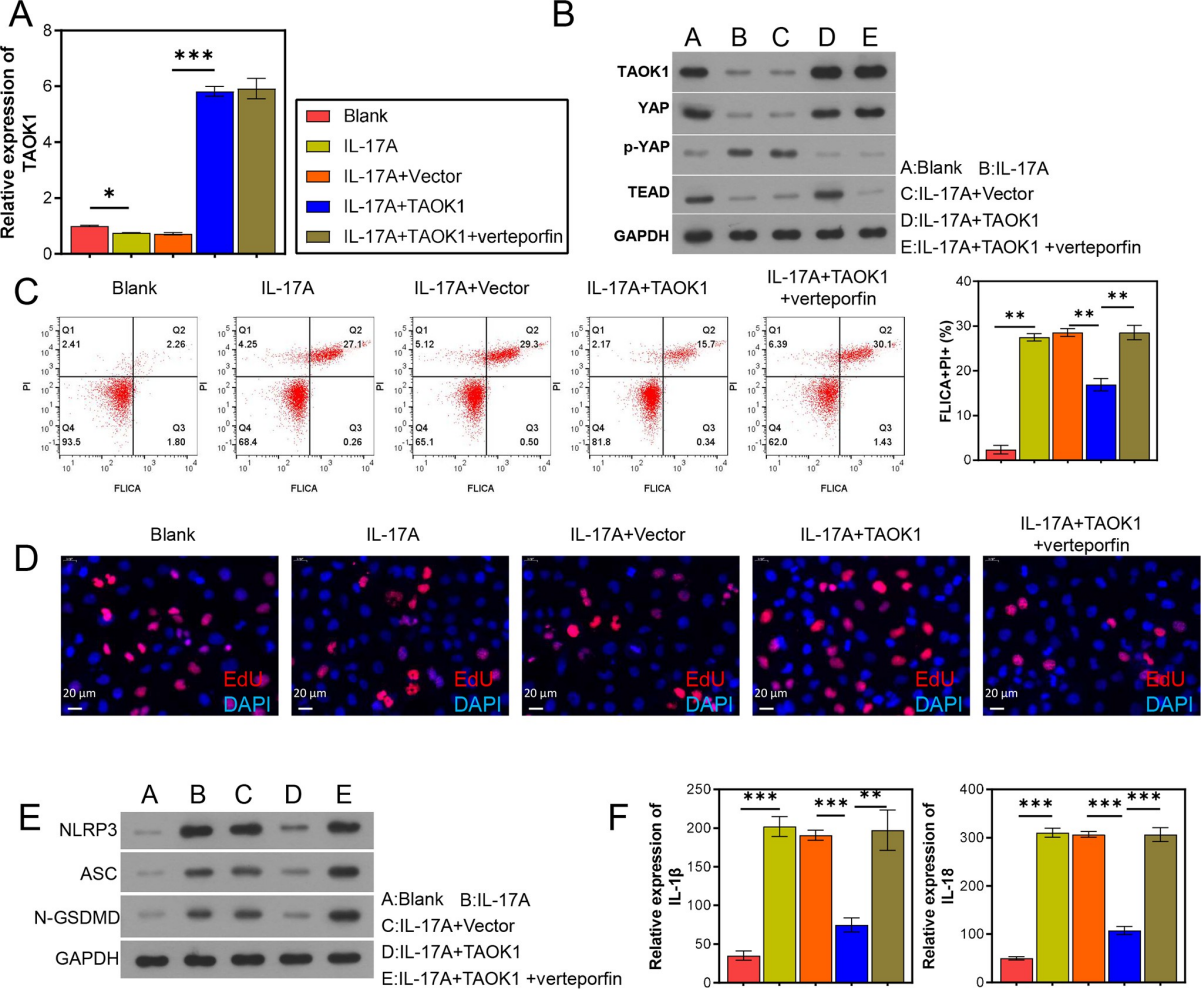
TAOK1 inhibited NLRP3 inflammasome activation via the YAP/TEAD pathway in the H9C2 cells. The H9C2 cells were subjected to *TAOK1* overexpression and verteporfin treatment, and their pyroptosis, proliferative capacity, and gene and protein expression levels were determined. (A, B) QRT-PCR (A) and WB (B) analyses were used to detect TAOK1 expression and YAP/TEAD pathway activation in the H9C2 cells. (C) FC analysis was used to detect the pyroptosis of H9C2 cells. (D) EdU staining assay was used to detect the proliferative capacity of H9C2 cells. (E) WB assay was used to detect the expression of NLRP3 inflammasome-related proteins in the H9C2 cells. (F) ELISA was used to detect IL-1β and IL-18 levels in the supernatant of H9C2 cells. *P < 0.05, **P < 0.01, ***P < 0.001.

To further clarify the regulatory role of TAOK1 on the YAP/TEAD pathway in the IL-17-treated H9C2 cells, the cells were administered verteporfin, and their proliferative capacity and NLRP3 inflammasome activation were observed. The results revealed that verteporfin treatment did not affect *TAOK1* expression in the IL-17-treated H9C2 cells (Fig 6A and 6B); however, it promoted the inhibitory effects of *TAOK1* overexpression on the proportion of FLICA+/PI+ IL-17-treated H9C2 cells and suppressed their proliferation (Fig 6C and 6D). In addition, verteporfin treatment upregulated NLRP3, ASC, and N-GSDMD levels, as well as IL-1β and IL-18 levels in the IL-17-treated H9C2 cells (Fig 6E and 6F). Furthermore, verteporfin treatment inhibited the YAP/TEAD pathway in the IL-17-treated H9C2 cells (Fig 6B).

### 3.6 TAOK1 regulated the activation of NLRP3 inflammasomes and the YAP/TEAD pathway *in vivo*

**Fig 7:**
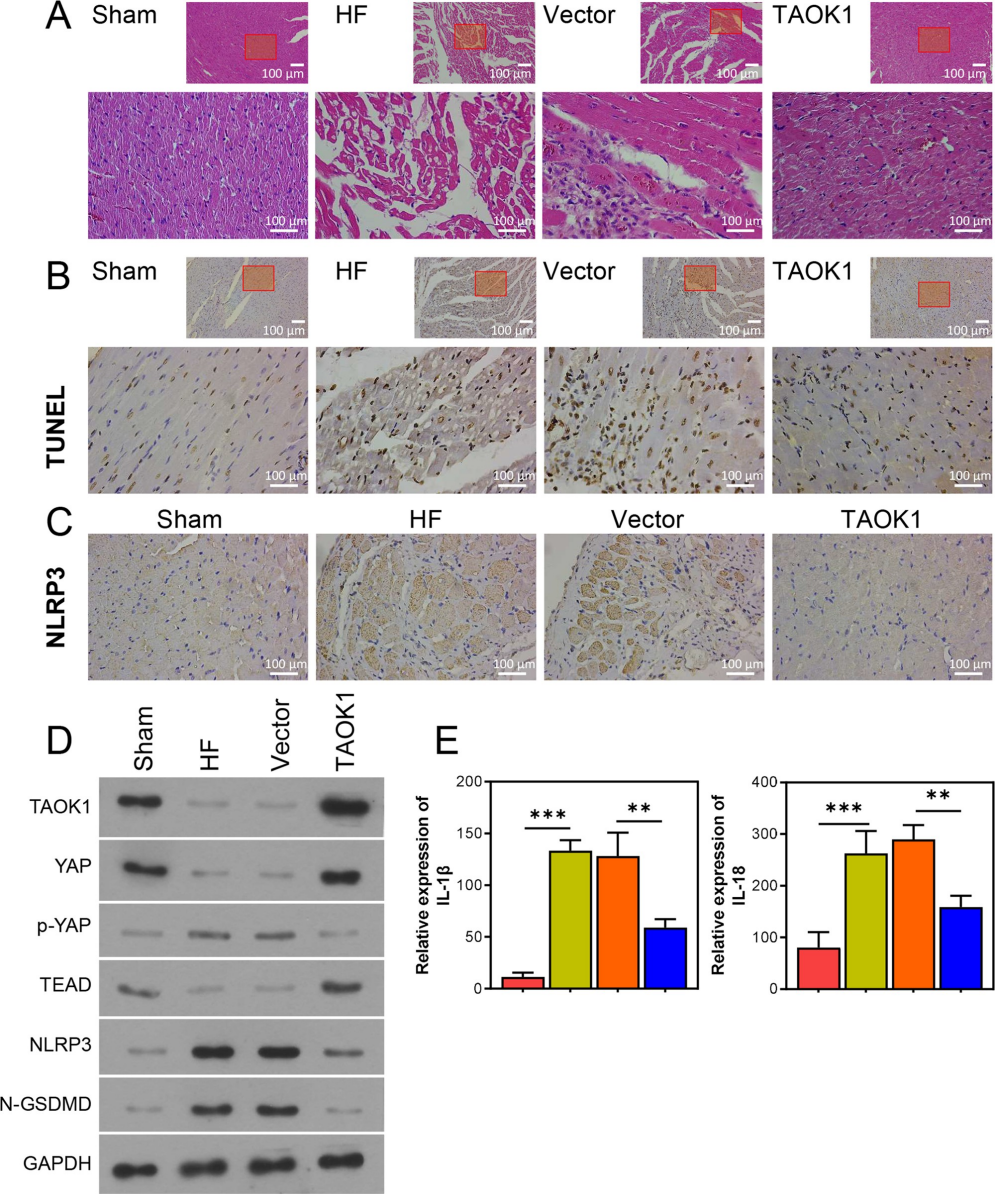
TAOK1 attenuated myocardial injury by suppressing the activation of NLRP3 inflammasomes and the YAP/TEAD pathway. The doxorubicin-induced HF rats were subjected to *TAOK1* overexpression, and their tissue pathology and protein expression levels were determined. (A) H&E staining was used to assess pathological changes in the myocardial tissues of HF rats. (B) TUNEL assay was used to detect myocardial injury in HF rats. (C) Immunohistochemistry was used to assess the intensity of NLRP3 expression in the myocardial tissues of HF rats. (D) WB assay was used to detect the expression of NLRP3 inflammasome- and YAP/TEAD pathway-related proteins in the myocardial tissues of HF rats. (E) ELISA was used to detect the serum IL-1β and IL-18 levels of HF rats. *P < 0.05, **P < 0.01, ***P < 0.001.

In this study, we established doxorubicin-induced HF rats and subjected them to *TAOK1* overexpression. The results revealed that doxorubicin treatment significantly altered the myocardial tissue and enhanced myocardial injury in the HF rats; however, these changes in the cellular pathology were significantly attenuated by *TAOK1* overexpression (Fig 7A and 7B). In addition, doxorubicin treatment significantly upregulated the expression of NLRP3, ASC, N-GSDMD, IL-18, and IL-1β and inhibited the YAP/TEAD pathway in the myocardial tissues of the HF rats; however, these changes were reversed by *TAOK1* overexpression (Fig 7C–7E).

## 4 Discussion

IL-17A, the prototype member of the IL-17 family, is a crucial cytokine that executes its biological activities by interacting specifically with IL-17 receptor A [29]. It has been found to exert a substantial influence on the progression of various cardiac diseases, such as dilated cardiomyopathy, autoimmune myocarditis, and myocardial infarction, indicating its crucial role and therapeutic potential in cardiovascular health [6, 30]. Recent research has demonstrated that IL-17A can have an adverse impact on the heart function of HF patients [10]. Our study found that IL-17A decreased cell proliferation and increased pyroptosis of H9C2 cells, further verifying the adverse effects of IL-17A on cardiomyocytes.

Recent studies identified TAOK1 as a suppressor of IL-17-induced signal transduction and inflammation [17]. However, the role TAOK1 in HF remains to be investigated. *TAOK1* knockdown enhances IL-17-induced cytokine and chemokine expression and NF-κB activation, which contribute to inflammatory responses [17]. In this study, we first examined the TAOK1 expression in H9C2 cells following treatments with varying concentrations of IL-17. While, further *TAOK1* overexpression reversed the IL-17-induced decrease in the pyroptosis of H9C2 cells, presenting a protective effect on cardiomyocytes. These results indicate the therapeutic potential of TAOK1 in HF treatment. Moreover, considering the regulatory role of TAOK1 on NF-κB, an upstream factor of NLRP3, our results suggest that NLRP3-mediated inflammasome may play a crucial role in the IL-17-mediated adverse effects on H9C2 cells.

NLRP3 inflammasome is an essential intracellular multiprotein complex and plays an important role in driving inflammatory responses in cardiovascular diseases [31]. The NLRP3 protein can assemble an inflammasome by recruiting ASC and pro-caspase-1 through its N-terminal PYD structural domain, resulting in the production of active caspase-1, which in turn processes cytokines such as pro-IL-18 into mature secretory forms, ultimately leading to pyroptosis [32]. Prolonged activation of the NLRP3 inflammasome is a primary contributor to tissue scarring, while activated inflammasomes can expedite the cardiovascular pathological process [20]. NLRP3 inflammasome-mediated inflammation is activated in doxorubicin-treated H9C2 cells [33]. Oral inhibitors targeting the NLRP3 inflammasome have been considered as prospective therapeutic agents for the treatment of cardiovascular diseases. Research has indicated that inhibiting NLRP3 inflammasome activation slows cardiovascular aging and telomere shortening, preserves cardiac function, and extends the lifespan of aged mice, suggesting that NLRP3 inflammasome has a regulatory effect on HF progression [34]. In our study, IL-17 treatment induced NLRP3 inflammasome activation in the H9C2 cells and led to an increase in their pyroptosis and a decrease in their proliferation capacity. However, *TAOK1* overexpression reversed the adverse effects of IL-17 on H9C2 cells, indicating their therapeutic potential in HF.

YAP plays a crucial role in cardiac development, hypertrophy, and HF. It contributes to the regulation of cardiomyocyte proliferation and cardiac development, and its dysregulation can lead to congenital heart diseases [35]. Under stress conditions, YAP activation can initially induce cardiac hypertrophy, followed by HF [36]. YAP activity is elevated in HF models, and its inhibition attenuates cardiac dysfunction and remodeling in HF [37]. The YAP signaling pathway can activate the NLRP3 inflammasome pathway. For example, IL-37 has been found to regulate macrophage programming through the YAP/NLRP3 pathway, indicating its therapeutic potential in myocardial infarction [38]. However, the role of YAP in the regulation of cardiomyocytes is complex. Lin et al. [39] found that the YAP/TEAD pathway is crucial for maintaining the normal functions of cardiomyocytes. Recent findings indicate that the YAP pathway is essential in ischemic heart injuries by maintaining cardiomyocyte proliferation [40]. Additionally, the YAP pathway has been associated with the mechanistic downregulation of inflammatory responses [41]. In this study, the YAP pathway was suppressed and the NLRP3 inflammasomes were activated in the *in vivo* and *in vitro* HF models. However, treatment with a YAP inhibitor attenuated the inhibitory effects of TAOK1 on the NLRP3 inflammasome-mediated pyroptosis. These results indicate the regulatory role of the YAP/TEAD pathway in the NLRP3 inflammasome-mediated pyroptosis.

## 5 Conclusions

Our study found that IL-17A concentration was positively correlated with the pyroptosis and negatively correlated with the proliferation and TAOK1 expression of H9C2 cells. Additionally, our results revealed that IL-17A activated the NLRP3 inflammasome by suppressing the YAP/TEAD pathway. However, TAOK1 overexpression reversed the IL-17A-induced pathological changes in HF rats by suppressing NLRP3 inflammasome-mediated pyroptosis and activating the YAP/TEAD pathway in cardiac tissues. Therefore, our study highlights the crucial role of TAOK1 in cardiac physiology and offers valuable insights into its therapeutic potential in HF.

## Supporting information

S1 Raw image (ZIP)
