# Supplementary figures and images for: TAOK1-mediated regulation of the YAP/TEAD pathway as a potential therapeutic target in heart failure
Source: PLoS One. 2024 Aug 9;19(8):e0308619. doi: 10.1371/journal.pone.0308619 (PMC11315341; doi:10.1371/journal.pone.0308619)

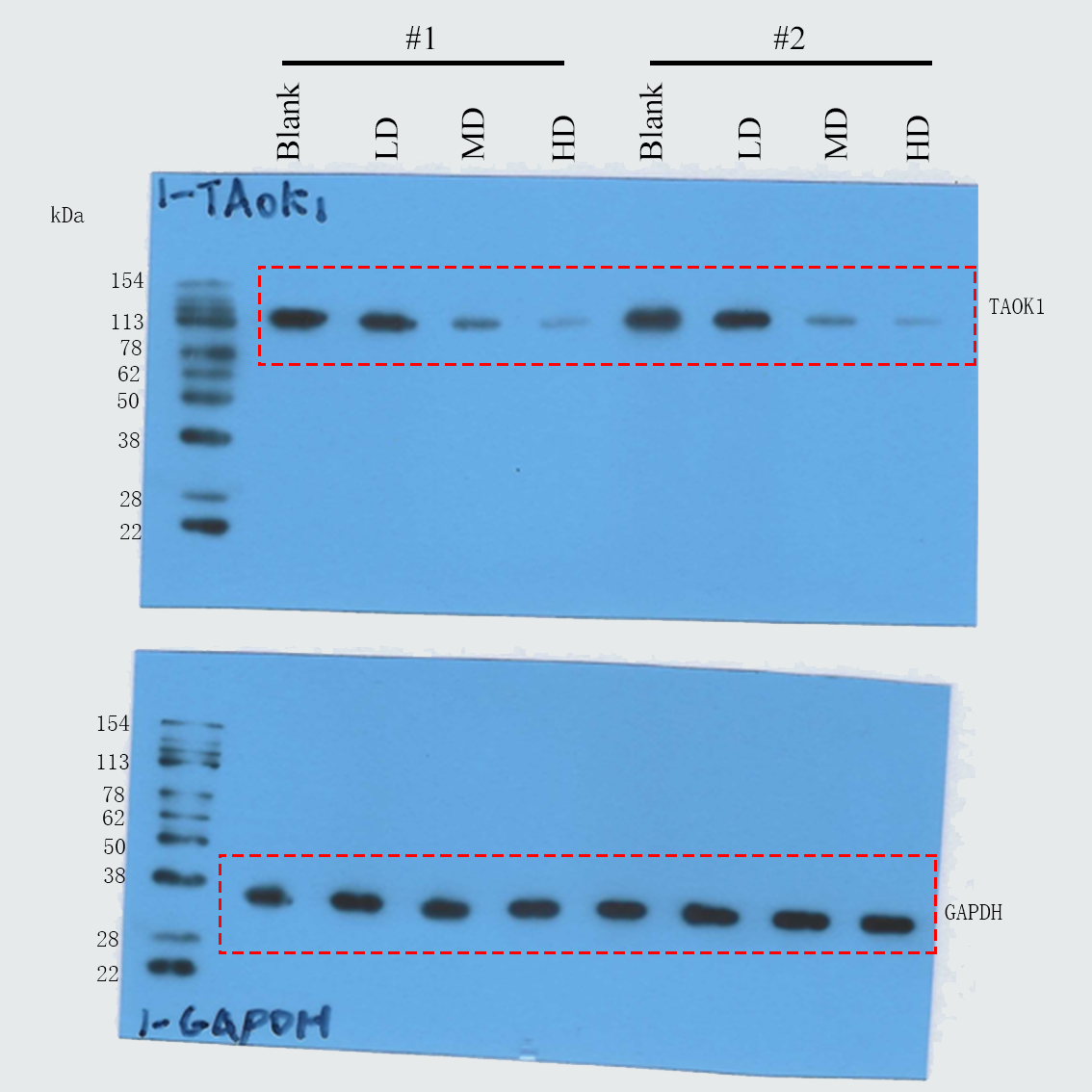

Supplement: S1 Raw image — (ZIP) [file pone.0308619.s001.zip › Fig2E.tif]

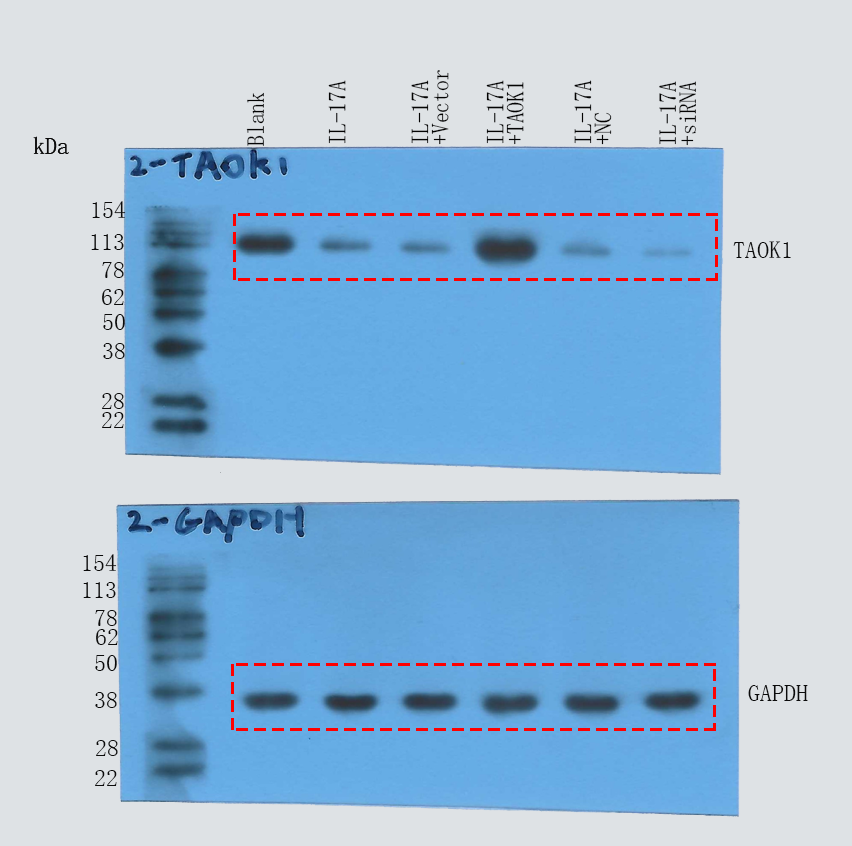

Supplement: S1 Raw image — (ZIP) [file pone.0308619.s001.zip › Fig3B.tif]

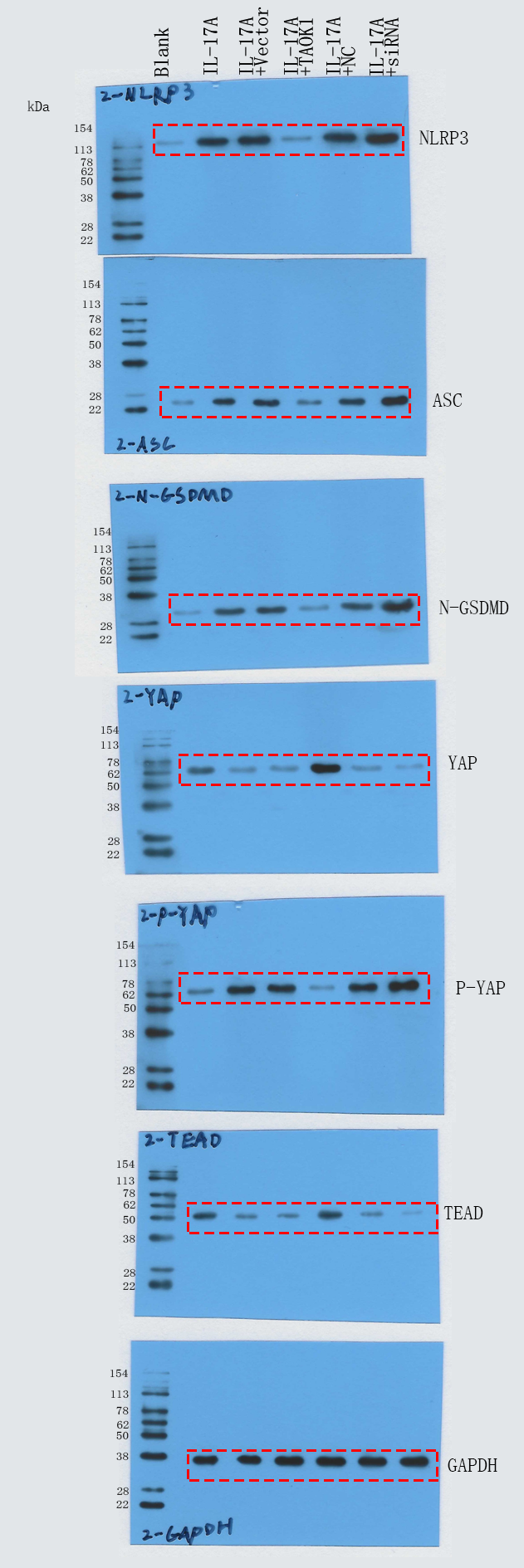

Supplement: S1 Raw image — (ZIP) [file pone.0308619.s001.zip › Fig3G.tif]

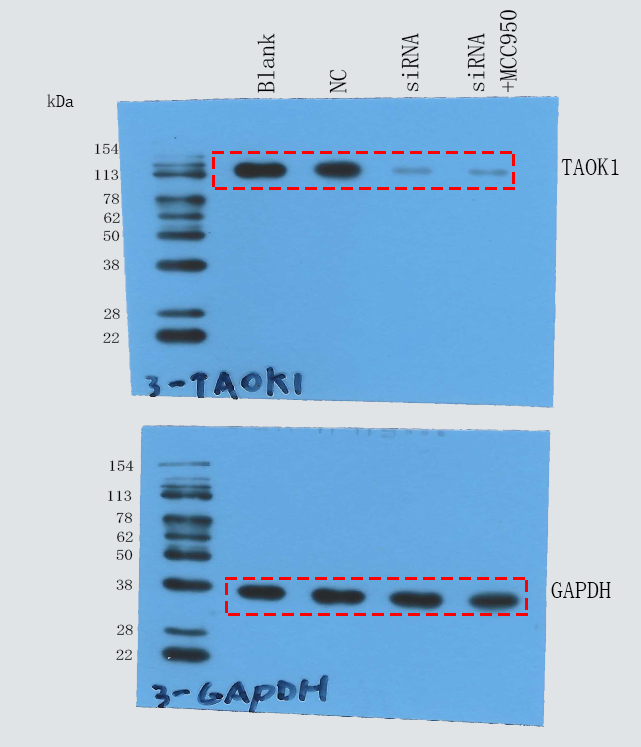

Supplement: S1 Raw image — (ZIP) [file pone.0308619.s001.zip › Fig4A.tif]

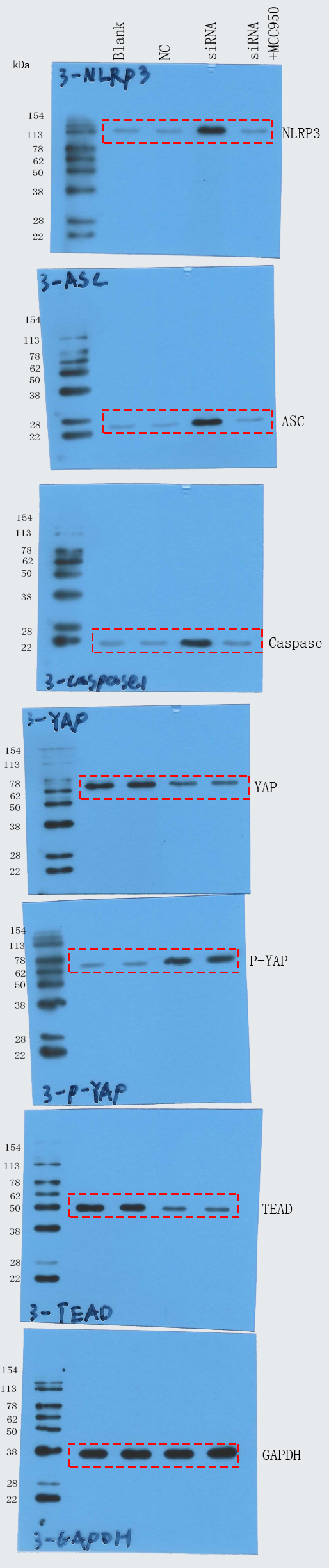

Supplement: S1 Raw image — (ZIP) [file pone.0308619.s001.zip › Fig4C.tif]

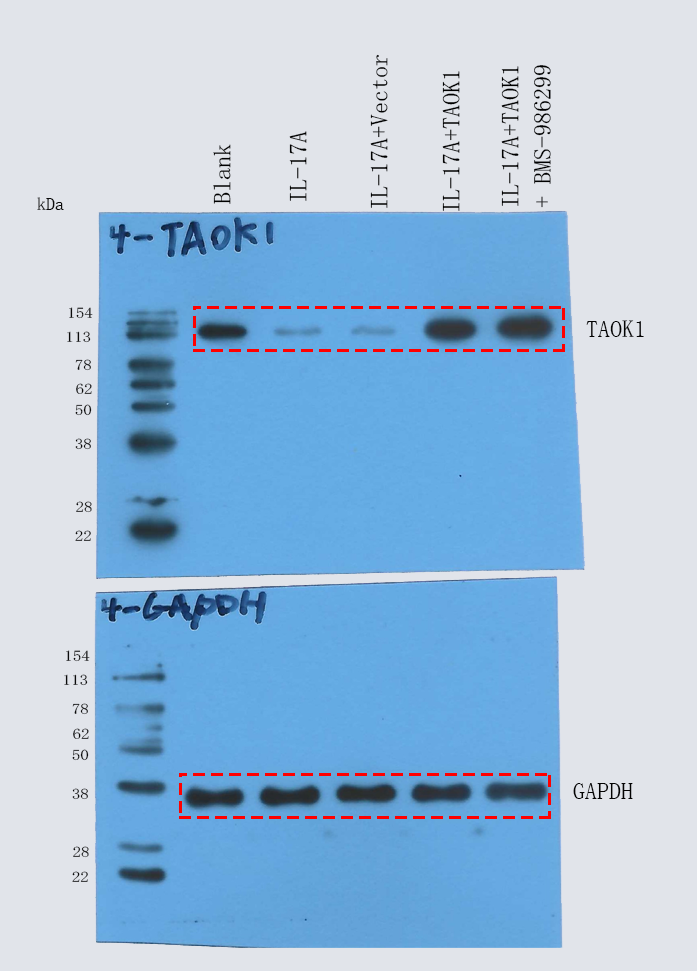

Supplement: S1 Raw image — (ZIP) [file pone.0308619.s001.zip › Fig5B.tif]

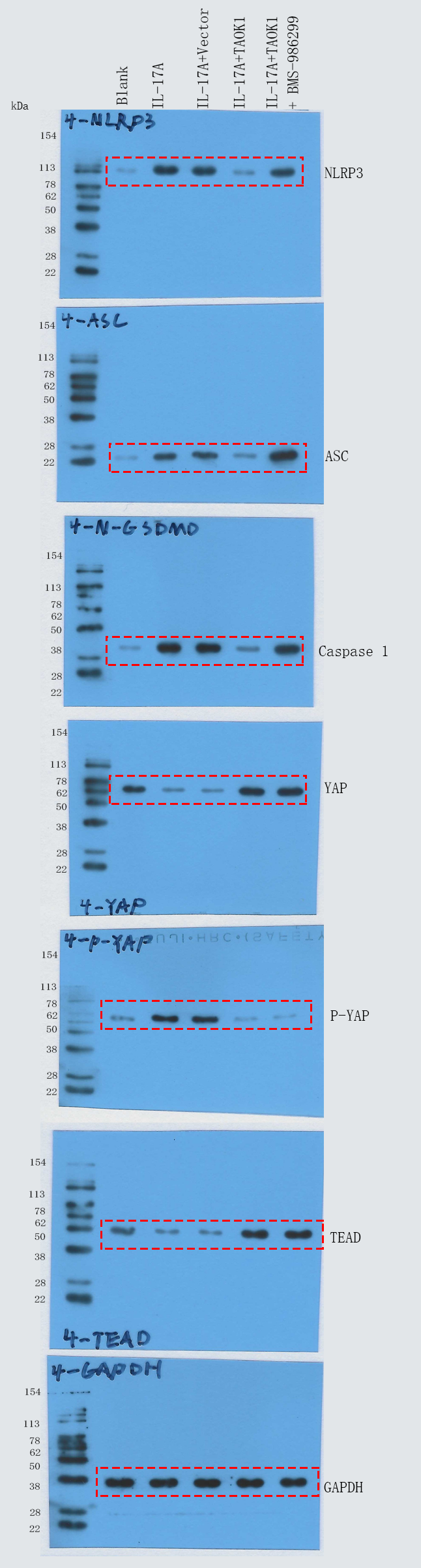

Supplement: S1 Raw image — (ZIP) [file pone.0308619.s001.zip › Fig5E.tif]

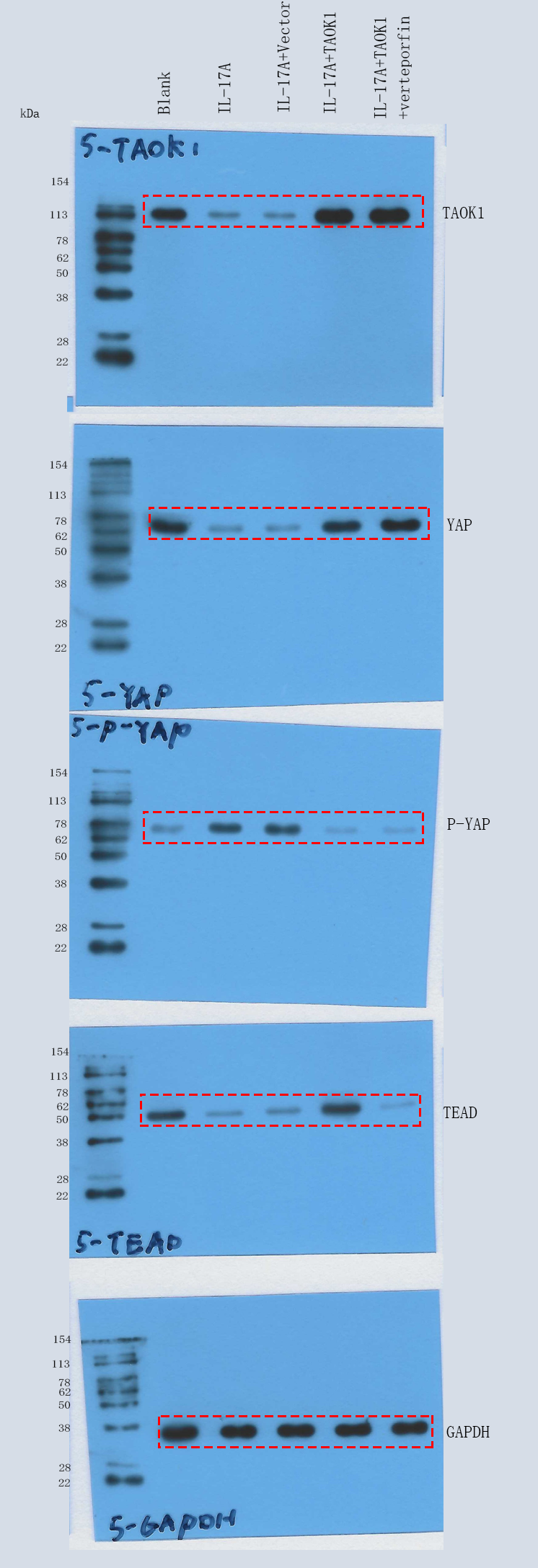

Supplement: S1 Raw image — (ZIP) [file pone.0308619.s001.zip › Fig6B.tif]

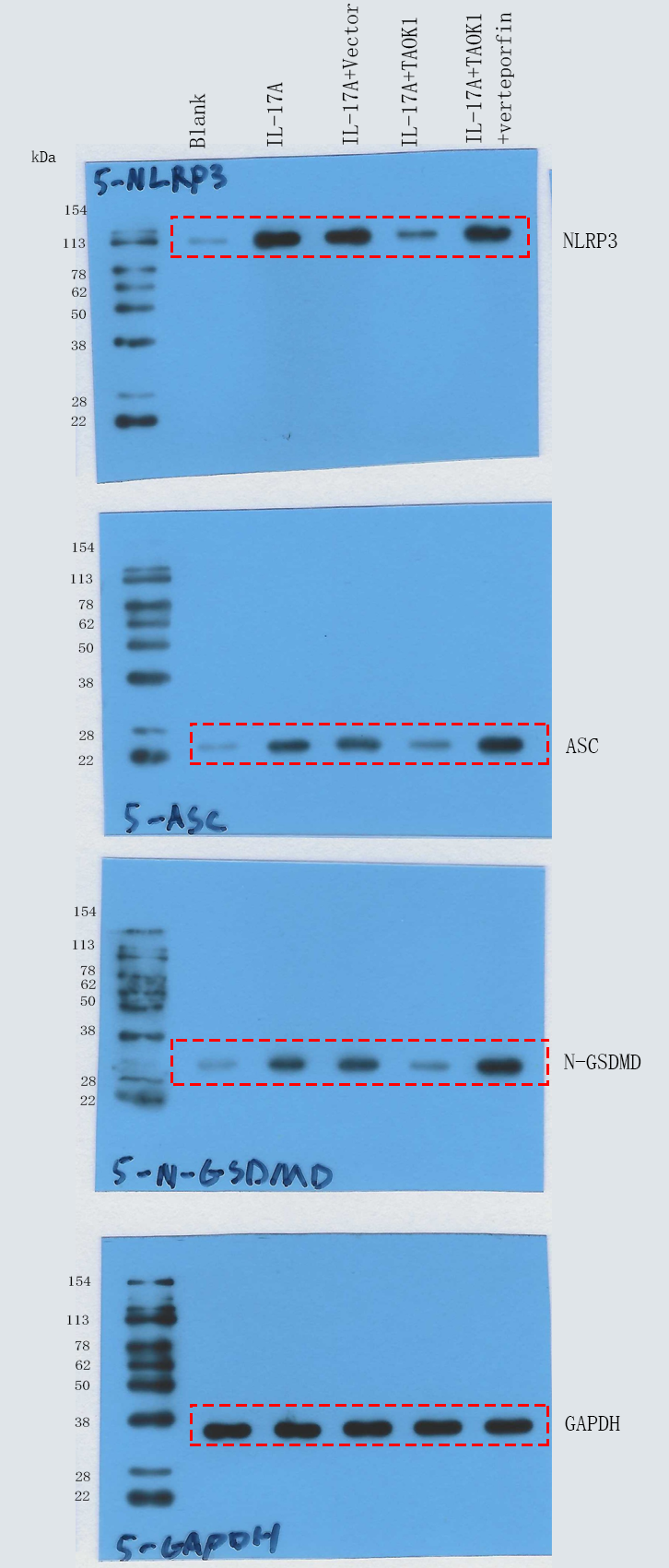

Supplement: S1 Raw image — (ZIP) [file pone.0308619.s001.zip › Fig6E.tif]

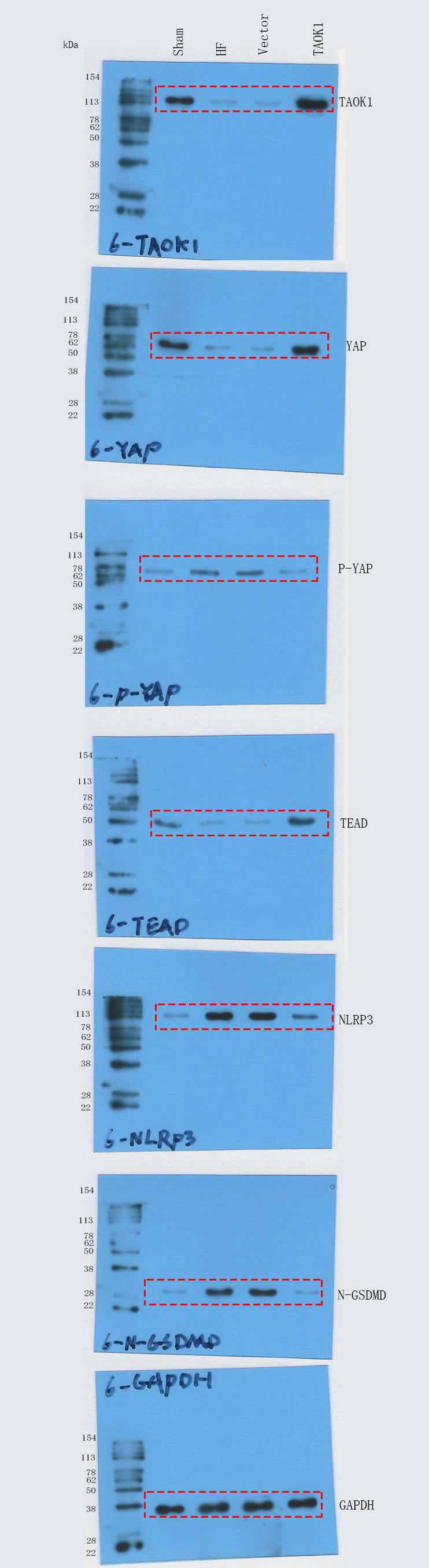

Supplement: S1 Raw image — (ZIP) [file pone.0308619.s001.zip › Fig7D.tif]
